# Very-high-power, ultra-short-duration radiofrequency for superior vena cava isolation: translational validation in porcine and clinical cohorts

**DOI:** 10.1093/europace/euag026

**Published:** 2026-03-05

**Authors:** Masaki Honda, Masateru Takigawa, Iwanari Kawamura, Tasuku Yamamoto, Miho Negishi, Ryo Tateishi, Kentaro Goto, Takuro Nishimura, Kazuya Yamao, Susumu Tao, Takehiro Iwanaga, Sayaka Suzuki, Iichiroh Onishi, Shinsuke Miyazaki, Tetsuo Sasano

**Affiliations:** Department of Cardiovascular Medicine, Institute of Science Tokyo, Yushima 1-5-45, Bunkyo-ku, Tokyo 113-8510, Japan; Department of Cardiovascular Medicine, Institute of Science Tokyo, Yushima 1-5-45, Bunkyo-ku, Tokyo 113-8510, Japan; Department of Cardiovascular Medicine, Institute of Science Tokyo, Yushima 1-5-45, Bunkyo-ku, Tokyo 113-8510, Japan; Department of Cardiovascular Medicine, Institute of Science Tokyo, Yushima 1-5-45, Bunkyo-ku, Tokyo 113-8510, Japan; Department of Cardiovascular Medicine, Institute of Science Tokyo, Yushima 1-5-45, Bunkyo-ku, Tokyo 113-8510, Japan; Department of Cardiovascular Medicine, Institute of Science Tokyo, Yushima 1-5-45, Bunkyo-ku, Tokyo 113-8510, Japan; Department of Cardiovascular Medicine, Institute of Science Tokyo, Yushima 1-5-45, Bunkyo-ku, Tokyo 113-8510, Japan; Department of Cardiovascular Medicine, Institute of Science Tokyo, Yushima 1-5-45, Bunkyo-ku, Tokyo 113-8510, Japan; Department of Cardiovascular Medicine, Institute of Science Tokyo, Yushima 1-5-45, Bunkyo-ku, Tokyo 113-8510, Japan; Department of Cardiovascular Medicine, Institute of Science Tokyo, Yushima 1-5-45, Bunkyo-ku, Tokyo 113-8510, Japan; Animal Research Facilities, Bioscience Center, Research Infrastructure Management Center, Institute of Science Tokyo, Tokyo, Japan; Japan Small Animal Medical Center, Saitama, Japan; Department of Pathology, Institute of Science Tokyo, Tokyo, Japan; Department of Cardiovascular Medicine, Institute of Science Tokyo, Yushima 1-5-45, Bunkyo-ku, Tokyo 113-8510, Japan; Division of Advanced Arrhythmia Research, Institute of Science Tokyo, Tokyo, Japan; Department of Cardiovascular Medicine, Institute of Science Tokyo, Yushima 1-5-45, Bunkyo-ku, Tokyo 113-8510, Japan

**Keywords:** Superior vena cava isolation, Very high-power ultra-short-duration ablation, Phrenic nerve injury

## Introduction

Superior vena cava isolation (SVCI) is an adjunct to pulmonary vein isolation (PVI) in patients with atrial fibrillation (AF) who have documented SVC triggers but it carries a high risk of phrenic nerve injury (PNI).^1,2^ Very-high-power short-duration (vHPSD; 90 W for 4 s) delivers brief, temperature-controlled energy that produces shallower but wider lesions.^3^ The multicenter peQasus study showed that temperature-guided HPSD/vHPSD enables safe, effective PVI in routine practice.^4^ Recently, pulsed-field ablation (PFA) has emerged as a non-thermal alternative with favourable PVI outcomes;^5^ however, transient sinus node dysfunction after SVCI using PFA has been reported,^6^ so radiofrequency remains the mainstay. Building on this, we evaluated an even shorter energy-delivery paradigm—very-high-power ultra-short-duration (vHPuSD; 90 W for 2–4 s per application)—and assessed acute safety and efficacy in a porcine model and a clinical cohort.

## Methods

### Study design

**Figure 1:**
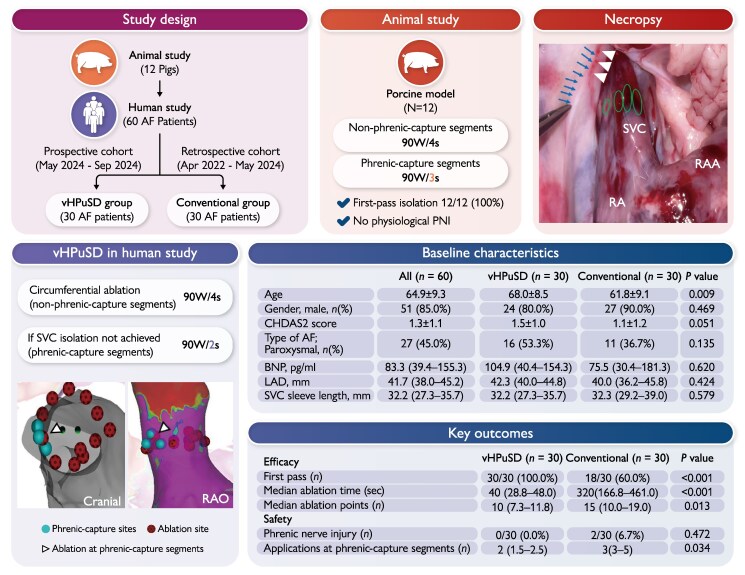
(*A*) Study design. Flowchart of the two-part study: an initial in vivo porcine study (*n* = 12) followed by a clinical SVCI study in 60 AF patients. The clinical arm comprised a prospective vHPuSD group (*n* = 30; May–September 2024) and a retrospective conventional group (*n* = 30; April 2022–May 2024). AF, atrial fibrillation; SVCI, superior vena cava isolation; vHPuSD, very-high-power ultra-short-duration. (*B*) Animal study. Encircling lesions on the SVC side were created with 90 W/4 s at non-phrenic-capture segments and 90 W/3 s at phrenic-capture segments. First-pass isolation was achieved in 12/12 (100%) with no physiological PNI. SVC, superior vena cava; PNI, phrenic nerve injury. (*C*) Necropsy. Representative gross view after SVCI. Ovals mark ablation sites on the SVC; arrowheads indicate the phrenic nerve; arrowheads denote mild discolouration of the contralateral pericardium along the phrenic nerve (observed in 8/12 animals). RA, right atrium; RAA, right atrial appendage. (*D*) vHPuSD in humans. Circumferential ablation of non-phrenic-capture segments at 90 W/4 s; if isolation was not achieved, phrenic-capture segments received 90 W/2 s. 3D reconstructions show phrenic-capture sites (spheres), ablation tags (dots), and lesions delivered at phrenic-capture segments (triangles). Abbreviations: RAO, right anterior oblique. (*E*) Baseline Characteristics. CHADS₂, congestive heart failure–hypertension–age ≥75–diabetes–stroke/TIA score; BNP, B-type natriuretic peptide; LAD, left atrial diameter. (*F*) Key outcomes.

This two-part study comprised a porcine model (*n* = 12) and a clinical cohort (*n* = 60) (*Figure 1A*). The clinical arm included a prospective vHPuSD group (*n* = 30; May–September 2024) and a retrospective conventional group (*n* = 30; April 2022–May 2024), and all underwent first-time SVCI after PVI, forming an observational, non-randomized comparison.

### Animal study

Porcine vHPuSD model (*n* = 12): non-phrenic-capture segments were ablated at 90 W for 4 s, and phrenic-capture segments at 90 W for 3 s (*Figure 1B*).

### Clinical procedures

SVCI was performed when the operator identified either documented SVC triggers or prominent SVC sleeves suggestive of arrhythmogenicity. We used the CARTO 3 system with an OCTARAY mapping catheter and a QDOT MICRO ablation catheter. vHPuSD applications were delivered with an irrigation flow of 8 mL/min and a temperature target of 60°C. Because applications were very short, catheter stability was confirmed before each application by fluoroscopy, 3D mapping, and contact force ≤10 g. The SVC–RA junction was identified on OCTARAY electrograms as the most cranial site with a typical right atrial near-field electrogram; the isolation line was created 5–10 mm cranial to it. Phrenic-capture segments were identified by 5 V/2 ms pacing along the lateral SVC. Ablation first targeted non-phrenic-capture segments at 90 W/4 s. If SVCI was incomplete, phrenic-capture segments received 90 W/2 s (*Figure 1D*). Diaphragmatic motion was assessed during and immediately after each application. PNI was further assessed by day-1 chest radiography and symptom review for dyspnoea. Procedural terminology and precautions followed the 2024 EHRA/HRS/APHRS/LAHRS AF ablation consensus.^7^

### Definitions and analysis

First-pass SVCI was defined as bidirectional block (entrance/exit) without any unplanned additional ablation. Continuous variables were compared with the Mann–Whitney *U* test and categorical variables with Fisher’s exact test; two-sided *P* < 0.05 was considered significant. Efficiency endpoints were first-pass SVCI, total RF time for SVCI, and the number of applications. Safety endpoints included PNI.

## Results

### Animal study

All 12 porcine procedures achieved first-pass SVCI (100%) with no physiological PNI (*Figure 1B*). At necropsy, mild discolouration along the contralateral pericardial phrenic-nerve course was seen in 8/12 animals (*Figure 1C*). Histologic evaluation was performed in only one of the eight animals with discolouration and showed no evidence of PNI.

### Clinical cohort

Baseline characteristics were similar between groups except for age: the vHPuSD group was older (68.0 ± 8.5 vs. 61.8 ± 9.1 years; *P* = 0.009) (*Figure 1E*). vHPuSD achieved 100% first-pass SVCI vs. 60% with conventional ablation (*P* < 0.001) (*Figure 1F*). Total RF time was 40 s vs. 320 s (*P* < 0.001), and the number of applications was 10 vs. 15 (*P* = 0.013). No clinically overt PNI occurred in the vHPuSD group, whereas two transient PNI events (6.7%) occurred in the conventional group; sinus node dysfunction was not observed in either group. The number of applications at phrenic-capture segments was significantly lower with vHPuSD (2 vs. 3; *P* = 0.034).

## Discussion

### Primary finding

A vHPuSD-based SVCI strategy enabled highly efficient acute SVCI achieving 100% first-pass isolation, and no clinically overt PNI occurred with vHPuSD whereas transient PNI occurred with conventional ablation.

### Efficacy

In previous experimental work, vHPSD (90 W/4 s) produces longer and wider lesions than conventional ablation,^3^ facilitating efficient encirclement with fewer applications. In our vHPuSD protocol, the circumferential SVC line was constructed predominantly with 90 W/4 s applications, which likely minimized gap formation and touch-ups, yielding a higher first-pass isolation rate with fewer applications than conventional ablation.

### Safety

Parameter selection at phrenic-capture segments was guided by prior evidence and our preclinical validation. In our porcine model, first-pass SVCI was achieved without physiological PNI, though mild discolouration appeared along the contralateral pericardial phrenic-nerve course with 90 W for 3 s, suggesting a possible subclinical thermal effect. Previous HPSD and vHPSD reports have described PNI with longer application durations or greater thermal energy near the phrenic nerve.^8,9^ Based on these observations and biophysical considerations that shorter applications limit thermal exposure,^10^ RF duration at phrenic-capture segments was restricted to 90 W for 2 s. No clinically overt PNI was observed with this setting in the acute clinical assessment, although subclinical or delayed injury cannot be excluded.

### Limitations

First, this single-centre, observational, non-randomized study used retrospective controls and is subject to residual confounding, including temporal changes in operator experience and practice patterns. We assessed only acute safety and efficacy; neither mandatory remapping nor long-term rhythm follow-up was performed in animals or patients, so lesion durability and clinical outcomes remain uncertain. SVCI was performed at the operator’s discretion in patients with suspected SVC involvement, so the clinical cohort represents a selected SVCI population rather than all AF ablation. Phrenic nerve safety was assessed only by symptom review and day-1 chest radiography, without continuous phrenic pacing or longer-term imaging, so subclinical or delayed PNI may have gone undetected. In the animal model, histological assessment was performed in only one of eight animals with discolouration, so subclinical structural injury in the remaining animals cannot be excluded.

## Conclusion

vHPuSD enabled highly efficient acute SVCI—with 100% first-pass isolation and no clinically overt PNI in this acute assessment—while reducing RF time and application count compared with conventional ablation. The long-term durability of SVC isolation with this ultra-short strategy remains to be determined and should be assessed prospectively.

## Data Availability

The data that support the findings of this study are available on request from the corresponding author. The data are not publicly available owing to ethical restrictions.

## References

[1] Takigawa M, Takahashi A, Kuwahara T, Okubo K, Takahashi Y, Nakashima E et al Impact of non-pulmonary vein foci on the outcome of the second session of catheter ablation for paroxysmal atrial fibrillation. J Cardiovasc Electrophysiol 2015;26:739–46. 25845757 10.1111/jce.12681

[2] Miyazaki S, Usui E, Kusa S, Taniguchi H, Ichihara N, Takagi T et al Prevalence and clinical outcome of phrenic nerve injury during superior vena cava isolation and circumferential pulmonary vein antrum isolation using radiofrequency energy. Am Heart J 2014;168:846–53. 25458647 10.1016/j.ahj.2014.09.011

[3] Takigawa M, Kitamura T, Martin CA, Fuimaono K, Datta K, Joshi H et al Temperature- and flow-controlled ablation/very-high-power short-duration ablation vs conventional power-controlled ablation: comparison of focal and linear lesion characteristics. Heart Rhythm 2021;18:553–61. 33127542 10.1016/j.hrthm.2020.10.021

[4] Heeger C-H, Almorad A, Scherr D, Szegedi N, Seidl S, Baran J et al Temperature-guided high and very high-power short duration ablation for atrial fibrillation treatment: the peQasus multicentre study. Europace 2025;27:euae284. 39504572 10.1093/europace/euae284 PMC12187331

[5] Schmidt B, Bordignon S, Neven K, Reichlin T, Blaauw Y, Hansen J et al European real-world outcomes with pulsed field ablation in patients with symptomatic atrial fibrillation: lessons from the multi-centre EU-PORIA registry. Europace 2023;25:euad185. 37379528 10.1093/europace/euad185 PMC10320231

[6] Ollitrault P, Chaumont C, Font J, Manninger M, Conti S, Matusik PT et al Superior vena cava isolation using a pentaspline pulsed-field ablation catheter: feasibility and safety in patients undergoing atrial fibrillation catheter ablation. Europace 2024;26:euae160. 38875490 10.1093/europace/euae160 PMC11252500

[7] Tzeis S, Gerstenfeld EP, Kalman J, Saad EB, Shamloo AS, Andrade JG et al 2024 European Heart Rhythm Association/Heart Rhythm Society/Asia Pacific Heart Rhythm Society/Latin American Heart Rhythm Society expert consensus statement on catheter and surgical ablation of atrial fibrillation. Europace 2024;26:euae043. 38597857 10.1016/j.hrthm.2024.03.017

[8] Kusa S, Hachiya H, Sato Y, Hara S, Ohya H, Miwa N et al Superior vena cava isolation with 50 W high-power, short-duration ablation strategy. J Cardiovasc Electrophysiol 2021;32:1579–88. 10.1111/jce.15060 33949738

[9] Otsuka N, Okumura Y, Kurokawa S, Nagashima K, Wakamatsu Y, Hayashida S et al In vivo tissue temperatures during 90 W/4s–very high-power short-duration (vHPSD) ablation versus ablation index-guided 50 W–HPSD ablation: a porcine study. J Cardiovasc Electrophysiol 2023;34:369–78. 36527433 10.1111/jce.15782 PMC10107763

[10] Nakagawa H, Ikeda A, Sharma T, Govari A, Ashton J, Maffre J et al Comparison of in vivo tissue temperature profile and lesion geometry for radiofrequency ablation with high-power short-duration and moderate-power moderate-duration: effects of thermal latency and contact force on lesion formation. Circ Arrhythm Electrophysiol 2021;14:e009899. 34138641 10.1161/CIRCEP.121.009899

